# *GeneTools *– application for functional annotation and statistical hypothesis testing

**DOI:** 10.1186/1471-2105-7-470

**Published:** 2006-10-24

**Authors:** Vidar Beisvag, Frode KR Jünge, Hallgeir Bergum, Lars Jølsum, Stian Lydersen, Clara-Cecilie Günther, Heri Ramampiaro, Mette Langaas, Arne K Sandvik, Astrid Lægreid

**Affiliations:** 1 Department of Cancer Research and Molecular Medicine, Norwegian University of Science and Technology, Trondheim, Norway; 2 Department of Mathematical Sciences, Norwegian University of Science and Technology, Trondheim, Norway; 3 Department of Computer and Information Science, Norwegian University of Science and Technology, Trondheim, Norway; 4 Department of Medicine, St. Olav's University Hospital, Trondheim, Norway

## Abstract

**Background:**

Modern biology has shifted from "one gene" approaches to methods for genomic-scale analysis like microarray technology, which allow simultaneous measurement of thousands of genes. This has created a need for tools facilitating interpretation of biological data in "batch" mode. However, such tools often leave the investigator with large volumes of apparently unorganized information. To meet this interpretation challenge, gene-set, or cluster testing has become a popular analytical tool. Many gene-set testing methods and software packages are now available, most of which use a variety of statistical tests to assess the genes in a set for biological information. However, the field is still evolving, and there is a great need for "integrated" solutions.

**Results:**

*GeneTools *is a web-service providing access to a database that brings together information from a broad range of resources. The annotation data are updated weekly, guaranteeing that users get data most recently available. Data submitted by the user are stored in the database, where it can easily be updated, shared between users and exported in various formats. *GeneTools *provides three different tools: i) *NMC Annotation Tool*, which offers annotations from several databases like UniGene, Entrez Gene, SwissProt and GeneOntology, in both single- and batch search mode. ii) *GO Annotator Tool*, where users can add new gene ontology (GO) annotations to genes of interest. These user defined GO annotations can be used in further analysis or exported for public distribution. iii) *e*GOn, a tool for visualization and statistical hypothesis testing of GO category representation. As the first GO tool, *e*GOn supports hypothesis testing for three different situations (master-target situation, mutually exclusive target-target situation and intersecting target-target situation). An important additional function is an evidence-code filter that allows users, to select the GO annotations for the analysis.

**Conclusion:**

*GeneTools *is the first "all in one" annotation tool, providing users with a rapid extraction of highly relevant gene annotation data for e.g. thousands of genes or clones at once. It allows a user to define and archive new GO annotations and it supports hypothesis testing related to GO category representations. *GeneTools *is freely available through www.genetools.no

## Background

Microarray technology allows researchers to monitor transcript levels of thousands of genes in a single experiment [1]. Typically it confronts the researcher with vast amounts of numerical data as a starting point from which to begin to investigate how molecular mechanisms are involved in a specific biological setting. Typically, scientists have to manually query several resources/databases for information. Although these can be highly informative individually, the collection of available content would be more useful if provided in an integrated manner. High-throughput, automated annotation summaries can expedite this step and today several resources like Source [2], GeneCards [3] and NetAffx [4] already offer this.

In order to understand how cells function within a tissue, e.g. in a given state one can use data-driven methods, such as hierarchical clustering and self-organizing maps [5,6], which identify groups of genes with similar expression patterns. However, a complementary approach is to view data at the level of biological background knowledge such as a gene's involvement in a biological processes or pathway. The leading controlled vocabulary for such functional information is Gene Ontology (GO) [7]. Annotation of genes with GO terms creates a biological knowledge profile, in three layers dependent on the top-level GO branch used (biological process, molecular function or cellular component).

Several tools are suited for analysis of the GO hierarchy and for statistical evaluation of GO category representations between gene lists [8]. Comparisons of gene lists are important in order to answer questions such as "are genes involved in process P overrepresented among the total of differentially expressed genes in an experiment" or "does treatment A induce more genes involved in process P than treatment B?".

A potential problem using such tools, is that the existing annotation databases are incomplete and for most organisms only a subset of the known genes are functionally annotated [8]. Moreover, a major part of the available annotations e.g. those inferred from electronic annotations may be imprecise or incorrect.

The present paper describes *GeneTools*, a package of web-based tools for gene annotation. *GeneTools *is built on top of an underlying database that is updated on a weekly basis to provide information as recent as possible. The annotation data is accessible through two user interfaces, the *NMC Annotation Tool *which offers general functional annotation information in both single- and batch search mode, and the *e*GOn tool which can annotate, display and perform statistical hypothesis testing to assess the degree of similarity of GO category representation between different gene lists. An important function in *e*GOn is the possibility to filter on evidence codes. Also, additional user defined GO annotations can be added to the database through the *GO Annotator Tool *for use in further analysis. Another unique feature in *GeneTools *is that user submitted data is stored in the database and can be shared with other users.

Finally, a significant part of this paper deals with how the hypothesis testing for GO category representations is performed, which we think has been inadequately described for many other resources.

## Implementation

*GeneTools *is a web service. It runs on most web browsers, including IE 5.0 or higher, Netscape 7 or higher and Mozilla Firefox 1.0 or higher, and is platform-independent. *GeneTools *is implemented in the PHP programming language. We have chosen to implement this tool as a web service to make it as user-friendly as possible, as most of the users are not bioinformaticians able to perform programming. However, more advanced use of the service is possible as described later in this chapter.

*GeneTools *is the front-end of a MySQL database containing annotation data from the following publicly available resources: UniGene [9], EntrezGene [10] (including GOA [11], Proteome, MGD [12] and RDG [13] annotations), Gene Ontology [14], SwissProt [15], and HomoloGene [16]. Information from 64 organisms available through UniGene is included, but the most comprehensive information is available for human, rat and mouse genes. All these databases are stored as local copies, enabling quick access to the data in response to the user query. Since many of the resources on which *GeneTools *draws continuously change their information content, the *GeneTools *database is updated on a weekly basis to ensure that it contains the most up-to-date information, continuously updating the stored gene reporter lists. An automated process checks for updates of the outside databases, downloads these files, and populates database tables accordingly. This ensures that the connections between external databases made within *GeneTools *are as accurate as possible. Thus, both the mapping of clones to genes and the functional attributes associated with those genes are dynamic and current. All data and graphics from searches and analysis can be exported in various formats (txt, XML or as Excel files).

Due to the heterogeneous nature of annotation information, bioinformaticians and systems biology researchers may want to perform more high-level analysis than offered through our web service. We therefore offer an API solution, based on web services description language (WSDL), for external resources wishing to use data from our database. Typically new and important tools like Taverna [17] can easily utilize this system using SOPE/RPC. Currently our API solution is utilized by the Norwegian Microarray Consortium (NMC) which updates their local BASE (BioArray Software Environment) [18] servers with information from this database. Moreover, SciCraft [19], a general data analysis tool, uses data from the *GeneTools *database in its microarray data analysis tool box. We will also offer R code for the statistical testing in *e*GOn upon request. The structure of our *GeneTools *database is built so that it can be used in the future as part of local or external data warehouses.

## Results and discussion

### Inputs

**Figure 1:**
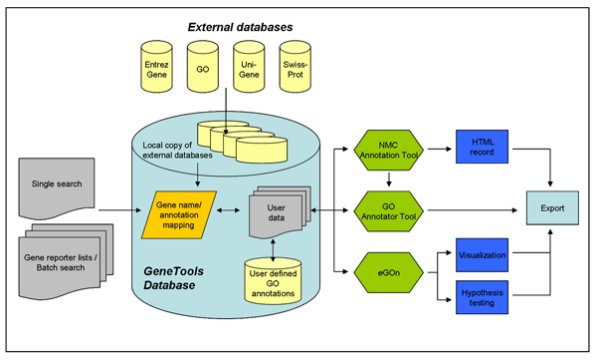
**Flowchart of the *GeneTools *program and the underlying database**. The underlying database is updated on a weekly basis with annotation information from several external databases including UniGene, Swiss-Prot, Entrez Gene and GO. User data are submitted to the database as text files of gene reporters and analysis of the annotation data can be performed through three user interfaces: the *NMC Annotation Tool*, the *GO Annotator Tool *and *e*GOn. Analysis results and annotation data can be exported in various formats.

Figure 1 gives an overview of *GeneTools *with its single search and batch search (gene reporter lists) inputs, its underlying database structure and associated tools for analysis. The ability to simultaneously collect data from numerous sources for e.g. thousands of genes from microarray experiments in batch is especially important and made very user friendly through *GeneTools*.

#### Single search

The database enables searching by gene symbols/names, GenBank accession numbers, UniGene cluster IDs, SwissProt entry names and several unique clone IDs (IMAGE clone IDs, University of Iowa clone IDs, Operon oligo IDs, TAIR IDs and a subset of selected Affymetrix and Agilent IDs).

The names and symbols of genes/proteins may be highly ambiguous [20]. We therefore recommend using primary gene IDs, like GeneBank accession numbers or specific probe IDs when querying the database. However, if gene names or symbols are used, caution is advised because only official names/symbols associated with UniProt knowledgebase will be recognized.

#### Batch search

Input of gene reporter lists for batch search is done by uploading tab-delimited text files to the server. After submission, the gene reporters are automatically mapped to a UniGene cluster, and functional annotations/attributes (e.g. GO annotation) are associated with the specific gene/protein (Figure 1). Uploaded gene reporter lists are stored and can easily be managed in folders or shared with other users. If new annotation information becomes available for any of the stored gene reporter lists, the user will be notified.

#### Updates

The user may at any time choose to update a stored gene reporter list, thus incorporating the most recent annotation information from the weekly update of the *GeneTools *database in the analysis. The updating process is fast even for lists of thousands of gene reporters. The user receives a specified report detailing which gene reporters are associated with new annotation information and the changes made.

### Tools, analyses and outputs

#### NMC annotation Tool

A major challenge when using genomic scale methods like microarrays, is to handle annotation information from the resulting comprehensive gene reporter lists. Thus, one of the most important features of *GeneTools *is the ability to simultaneously extract pre-existing annotation data from a wide variety of database resources for thousands of genes in a batch. Since the *GeneTools *database is weekly updated and the *NMC Annotation Tool *provides user friendly functionalities for associating new annotation information with the reporters in uploaded gene lists, the *NMC Annotation Tool *is particularly useful when it is important to always have access to the most recent information on the genes and clones being examined. The *NMC Annotation Tool *enables the user to query the *GeneTools *database by singe gene search or by batch search after submission of a gene reporter list for a microarray experiments. Given the massive amount of data available through *GeneTools *(Figure 1), information overload can be a potential problem. Therefore, we have provided the user with the option to select (in the "preferences" menu) the information to be shown on the screen for single search and batch view and to select which information to export. However, we will stress that this option should be used cautiously, because it may introduce selection bias and important information may be lost.

#### Single search outputs

The single search function captures the collection of features attributable to the given gene and its products, when a gene is defined by a unique UniGene cluster. Whenever available, each single search result view will contain all or a subset of the following categories of data:

I. Data from Unigene, including e.g. A. gene cluster, name and symbol. B. protein similarities with selected organisms (with direct link to Entrez protein). C. chromosome localization information. D. UniGene associated sequences with cluster.

II. Data from Homologene: Shows homologous genes for human, rat and mouse.

III. Data from Entrez Gene: A. gene name, symbol and aliases. B. biological roles and summary of functions curated by Entrez (Ref.seq summary). C. gene ontology (GO) annotations with direct link to references and links to alternative ontologies like KEGG. D. direct link to curated PubMed Gene RIFs (reference into function).

IV. Data from Swiss Prot: A. protein names and aliases. B. biological role and function information curated by Swiss Prot. C. protein sequence information. D. direct links to various external sources associated with current protein are offered for each gene reporter.

#### Batch search outputs

**Figure 2:**
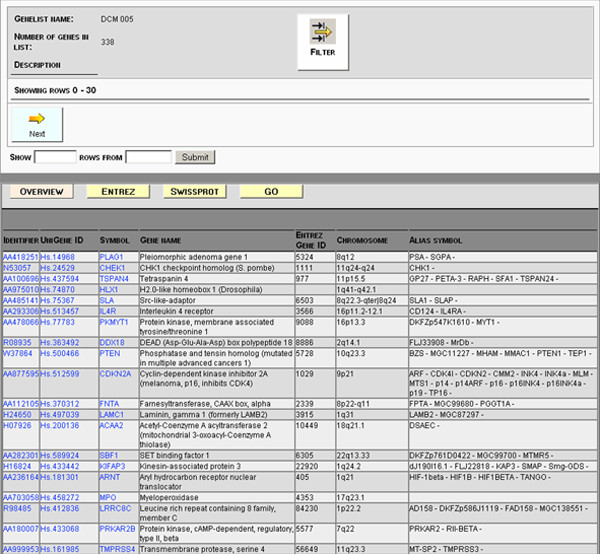
**Typical "overview" result output for a submitted gene reporter list**. Input gene reporter and associated UniGene cluster, gene name, symbol and chromosome localization is shown for all the gene reporters in the submitted lists. Several of the information boxes are hyperlinked redirecting the user to the original source. More specific annotations can be found under the "tabs" named Entrez, SwissProt and GO. By clicking on the gene reporter ID, a single search window for the selected gene reporter will appear.

One of the most important and unique features of the *NMC Annotation Tool *is the batch search mode which utilizes all of our database sources for gene reporter lists from microarray experiments. For instance, the users can easily extract biological function, chromosomal localization, and get access to publications (GeneRIFs) that describe gene functions. The results for reporter gene list from a batch search can be viewed in a user-friendly tabular form (Figure 2). Moreover, the annotation data displayed on the screen are associated with hyperlinks to the underlying database or to the single search view. The annotation data can be exported in several formats for printing or storage (XML and XLS).

*NMC Annotation Tool *provides several features not available in other gene annotation tools. To our knowledge, few other application stores users' gene reporter lists allowing update of the reporter lists at any time with the most recent UniGene, Entrez Gene and GO information. This is important since the clusters in UniGene change rapidly and new GO annotations are being added continuously. To achieve this, the submitted gene reporter lists can easily be updated with all new information. Information about the external databases included in *GeneTools *and their last updates can be found from a link named "database status" in the menu, and provides useful documentation for publishing purposes. Information about commercial arrays supported by *GeneTools *(currently Affymetrix, Operon and Agilent) is also given. To our knowledge, a similar variety of important features is not available in gene annotation tools like Source [2], GeneCards [3], NetAffx [4], GeneCruiser [21], Onto-Tools [22], GARBAN [23] and GeneLynx [24].

#### GO annotator tool (user defined GO annotations)

The introduction of Gene Ontology (GO) [25] as a standardised vocabulary for describing genes, gene products and their biological functions represents an important milestone in the possibilities to handle and include biological background information in functional genomics analyses. Many databases today provide GO annotations for a variety of organisms including humans and other species. However, GO is still incomplete and significant extensions to its structure are needed before all available biological knowledge can be represented as GO annotations in public databases. Also, besides the human research filed other organisms e.g. common model organisms like rat and mouse are still lagging behind when it comes to raising the quality of curation of GO annotations. Thus, a high proportion of GO annotations offered in the rat genome database (RGD) [13] and the mouse genome database (MGD) [12] are associated with the IEA (inferred by electronic annotation) evidence code, which implies a lower degree of certainty than some users may require.

**Figure 3:**
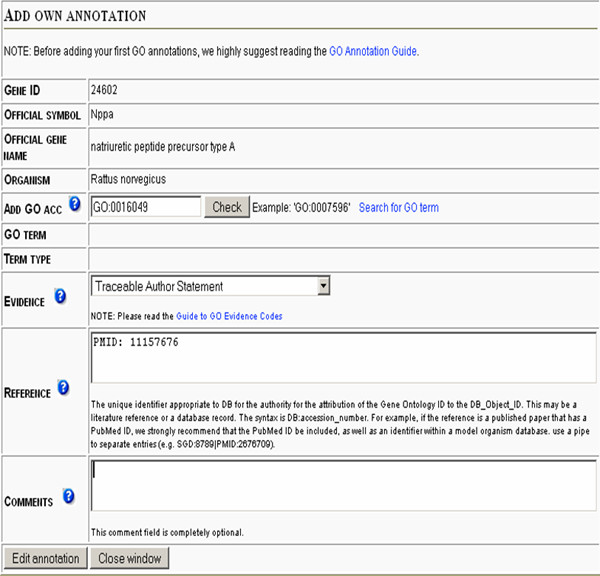
**User interface for the *GO Annotator Tool***. To add a new GO annotation, the user selects a gene, adds a GO term, chooses an appropriate evidence code and adds a reference article (PMID). The GO annotations are then stored in the database and an exported function to GOA for world wide distribution is under development. A link to the *GO Annotator Tool *can be launched from the top of the page of the result window from a single gene search, in the *NMC Annotation Tool *mode.

To overcome at least some of these problems, *GeneTools *allows a user to define their own GO annotations to genes of interest. The *GO Annotation Tool *(accessible through "single search" mode in the *NMC Annotation Tool*) enables the addition of new, user defined GO annotations as well as the curation of GO annotations e.g. annotations with evidence code IEA. *GO Annotation Tool *is supported by a GO term search system, simplifying the browsing for GO terms. Evidence codes and references (e.g. PMID) according to GO standards and free text can be added (Figure 3). New annotations are stored in the database and can be included in further analysis (e.g. added to the GO analysis in the *e*GOn tool). We are in the process of making an export function, where these user defined GO annotations can be exported to the GOA database [11] by an email service. GOA will curate these annotations and make them available for others through the GO annotation database [26].

#### Explore Gene Ontology (eGOn)

Controlled vocabularies facilitate query and retrieval of knowledge from many different sources using a common query structure. Three separate important activities are needed to enable this: the production and maintenance of the ontologies themselves; the creation of associations (or annotations) between the GO terms and gene products, and the development of tools that facilitate the creation, maintenance and use of the ontologies.

*e*GOn visualizes gene annotations in the GO hierarchy and offers a collection of statistical tests that translate the GO annotation information associated with the reporters in gene lists from functional genomics experiments to provide insight into the biological mechanisms involved.

A wide range of resources are available for GO analysis [27]. In a recent review, Khatri et al. [8] question how such resources are built and used. Khatri et al. point out that existing annotation databases are incomplete, that a proportion of the annotations may be imprecise or incorrect, that name space mapping (how to connect a probe sequence to a gene/protein) is a problem, and that available statistical tests are not always validated. We think that the tool *e*GOn of the *GeneTools *suite meets many of these challenges since it enables filtering of annotations by evidence code, it allows the entry of new annotations and curation via the *GO Annotator Tool *and it provides a series of robust statistical tests that are thoroughly validated and documented.

For GO annotations, *GeneTools *uses Entrez Gene which offers curated data from the GO database that includes all registered GO annotations [26]. Some annotations available in the GO database will not be included using the Entrez curated GO annotations but the quality of annotation is most likely better. *e*GOn offers the possibility to filter the GO annotations from a gene reporter list by evidence codes. A substantial proportion of GO annotations are inferred by electronic methods (evidence code IEA), potentially being imprecise and possibly biasing further analysis. Thus, in a given analysis, it may be beneficial to exclude IEA annotations and only use more robust annotations, like e.g. annotations derived from "traceable author statement" (TAS), "inferred from direct assay" (IDA) or "inferred by curator" (IC). In other situations it may be desirable to include electronic annotations in order to obtain a sufficient amount of data to do a valid analysis, e.g. for rat and mouse genes where most of the annotations up to now are IEA. Another possibility which to our knowledge is not in use by any GO analysis tool today, might be to perform some kind of weighting by the type of evidence code for the statistical calculations.

**Figure 4:**
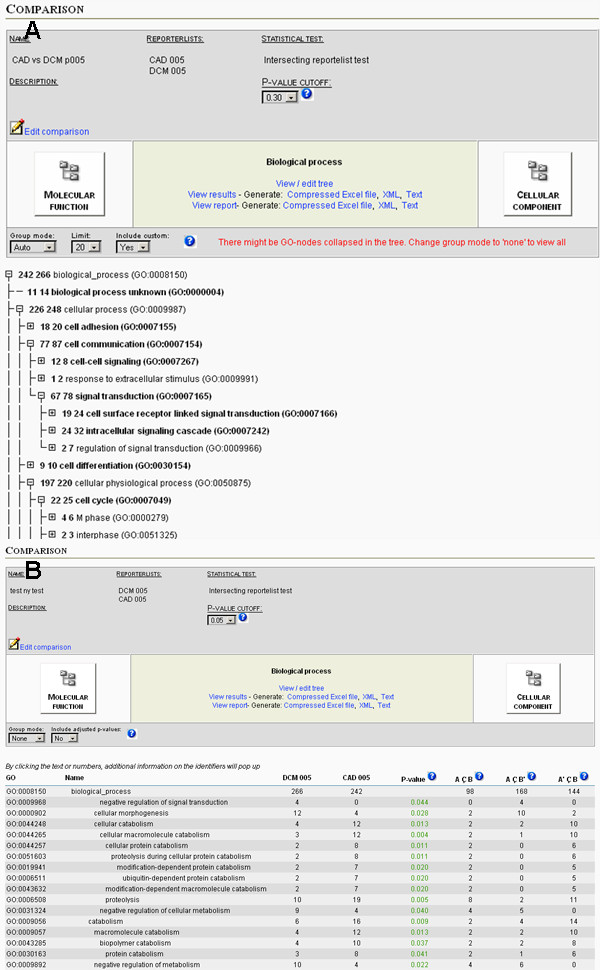
**Result report output from *e*GOn**. Gene reporter lists submitted to *e*GOn can be visualized in tree-view, as result-view or as report-view. In the tree-view (A) the nodes may be collapsed or expanded producing the desired level of detail and the resulting structure can be saved as a template for future use. Several preset levels can also be selected. By clicking on a GO node the gene reporter associated with this GO node in the GO-tree can be interactively examined and links are offered to single gene view in the *NMC Annotation Tool*. In result view p-values for all GO categories are shown and for the report view (B), only the GO categories that fit the user's p-value cut-off are shown.

An essential feature of *e*GOn is the possibility to compare and analyze annotated genes from two or more gene reporter lists in the GO-tree. *e*GOn both visualizes these comparisons within the GO-tree and formally calculates the degree of GO category representation similarity between the gene lists using statistical tests (Figure 4).

#### Testing statistical hypotheses of association between gene reporter lists

To investigate and better interpret the relevance of biological annotations of lists of gene reporters, statistical hypothesis testing can be a valuable tool. Let us for example consider a microarray experiment where the objective of the study is to compare the differentially expressed genes from heart failure tissue between cases and controls where the cases are patients with coronary artery disease (CAD) or dilated cardiomyopathy (DCM) and the controls are tissue from non-failing hearts [28].

To formally state the statistical hypothesis, consider a randomly chosen gene and a given GO category denoted G. Define the following three events:

• A = the gene is in gene reporter list A

• B = the gene is in gene reporter list B

• G = the gene is a member of GO category G.

In this example the list A would be the list of differentially expressed genes between CAD and controls while list B would be the differentially expressed genes between DCM and controls. At the given GO category G (e.g. catabolism), we are interested in investigating whether the probability of belonging to GO category G is different for genes on gene list A and genes on gene list B. For each gene on list A, there is a conditional probability P(G|A) of belonging to GO category G, and for each gene on list B, there is a conditional probability P(G|B) of belonging to GO category G. Under the null hypothesis these two probabilities are equal. From this the following null hypothesis and alternative hypothesis can be formulated.

H_0_: P(G|A) = P(G|B) vs. H_1_: P(G|A) ≠ P(G|B)

**Figure 5:**
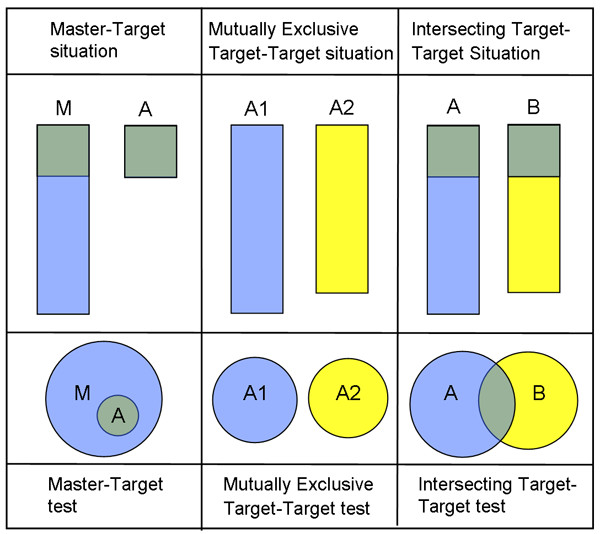
**Three different situations covered by the statistical testes in *e *GOn**. *Master-target situation*: When one gene reporter list is a subset of the other list (the master list) the master-target test can be used in the comparison. *Mutually exclusive target-target situation*: If the gene reporters do not have any reporters in common (e.g. lists of up- vs. down regulated genes form the same experiment) the mutually exclusive target-target test can be used. *Intersecting target-target situation*: if the two lists compared include common gene reporters, from e.g. two experiments, then the intersecting target-target test can be used.

By using the laws of conditional probability, we have the following additional interpretation. For a chosen GO category G, the ratio between the probability of membership of gene reporter list A and membership of gene reporter list B, is the same as the ratio between the probability of being a member of gene reporter list A to the probability of being a member of gene reporter list B in the whole GO-tree. Statistically we need to distinguish between three situations, to correctly handle the possible dependencies between gene reporter lists A and B. An illustration of these situations is given in figure 5. Different statistical hypothesis tests are suitable for the three situations. In *e*GOn we have implemented three tests for these situations: the master-target test, the mutually exclusive target-target test and the intersecting target-target test. In brief, all three tests are parametric and the tests for the master-target situation and the mutually exclusive target-target situation are based on the same implementation of Fisher's exact test, but with different inputs. The intersecting target-target test is based on a test statistic by Leisenring et al. [29]. The test of Leisenring is designed to test if the positive predictive value (PPV) of two medical diagnostic tests is equal. A further description of the different situations and the corresponding tests can be found in the next chapters. Moreover, a detailed description of the statistical tests is offered in the supplementary material (additional file 1).

#### Master-target situation

In the master-target situation the GO categories (e.g. biological processes) of the genes of interest (e.g. differentially expressed) from a given experiment (target list) are compared with the distribution of GO categories for all gene reporters represented as physical probes on the microarray (master list) used in the experiment. The purpose is to find whether, in any of the GO categories, the genes of interest are over- or underrepresented compared to the genes represented on the microarray. For our heart failure example, list M would be a list of all the genes investigated on the microarray and list B would be the genes that are found to be differentially expressed between the DCM hearts and the controls (Figure 5).

This type of comparison between two gene reporter lists is useful and most GO tools offer tests for this. Statistically this situation can be transformed into a problem where we for each GO category under consideration want to test if two independent binomial proportions are equal (for details, see Günter et al. [30]). Several statistical approaches can be used, e.g. Fisher's exact test, Pearson's asymptotic Chi-square-test, a conditional mid-p test, or an unconditional test. We refer to Agresti [31] for a presentation of these tests, and to Khatri and Dragici [8] for an overview of different statistical tests implemented in the various GO-tools available in the master-target situation. In *e*GOn we have chosen the Fisher's exact test for the master-target situation and we call this the master-target test. The implementation is based on a translation to PHP of a JAVA-script by Langsrud [32]. The use of this two sided test is further explained by Zeeberg et al. [33].

#### Mutually exclusive target-target situation

In the mutually exclusive target-target situation there are no common genes in the two lists compared, in the heart failure example list A1 could be the list of differentially expressed genes that are up-regulated for the CAD hearts compared to the controls, while list A2 contains the genes that are down-regulated for the CAD hearts compared to the controls. The purpose with this type of comparison is to find which e.g. biological processes as defined by GO categories are differentially represented in the up- and down-regulated genes in the same experiment (Figure 5).

Statistically this situation is very similar to the master-target situation and can be transformed into a problem where we for each GO category under consideration want to test if two independent binomial proportions are equal. The same statistical tests as listed for the master-target test can be used. In *e*GOn we have chosen to implement the Fisher's exact test for the mutually exclusive target-target situation, called the mutually exclusive target-target test, using the same implementation, but with different inputs, as for the master-target test.

#### Intersecting target-target situation

When two gene reporter lists are compared and a number of gene reporters are represented on both lists, the intersecting target-target test is used to investigate whether the GO categories represented by these genes are over- or under represented in the experiments behind the two lists. In our heart failure example, list A could be the differentially expressed genes between CAD hearts and controls while list B would be the differentially expressed genes between DCM hearts and controls (Figure 5).

In Günther et al. [30], three different statistical tests are presented in the situation where the two gene lists are intersecting. All three tests are constructed for use with large samples, and are based on an asymptotic relation to the Chi-square distribution. In *e*GOn we have chosen to implement the test based on Leisenring et al. [29], originally constructed for comparing positive predictive values of two diagnostic tests, tests A and B, with respect to a disease G. This test uses a score statistic based on generalized estimating equations to fit a generalized linear model. We have translated this test into the setting of comparing two gene lists at a given GO category. Further details can be found in Günther et al. [30] or in the supplementary material (additional file 1).

#### Methodical considerations

The statistical tests for association between two gene reporter lists under consideration are based only on the gene lists submitted to *e*GOn, and the raw data underlying the statistical analyses producing the gene reporter lists are not submitted to *e*GOn. This means that *e*GOn does not offer permutation based methods for addressing the dependence structure between the genes. The statistical tests in *e*GOn are thus based on the assumption that under the null hypothesis the genes on the lists (or subsets of the lists in the intersecting target-target situation) act independently, as is also commonly assumed in other GO-tools. This should be taken into consideration when analysis is performed, and duplicate genes/reporters, close family members or pathways partners may be removed. This can easily be done by the filtering tool in *GeneTools*.

The p-values produced by the statistical test can be displayed for all GO categories or only those satisfying a certain p-value cut-off. Adjusted p-values can be calculated for a selected set of GO categories and is dependent on how the GO hierarchy is collapsed/expanded, using the step-up procedure of Benjamini and Hochberg [34] for controlling the False Discovery Rate (FDR). Setting a cut-off at 0.05 for the adjusted p-value will control the (FDR) at level 0.05. The Benjamini-Hochberg step-up procedure controls the FDR under certain dependence structures (for example positive regression dependency, see Benjamini and Yekutieli [35] for a detailed presentation). However, the dependency structure among the selected GO-categories in the GO-tree is not known, and questions remain about controlling the FDR in hierarchical structures.

One important "consensus point" within statistical inference discussed by Allison et al. [36] is that gene set testing is desirable, and has become a popular and widely accepted analytical tool. However, one problem with gene class testing, according to Allison et al. [36], is that the null hypotheses of these tests are not, or poorly defined. By formally stating the null and alternative hypotheses, we think our paper has addressed these concerns in a thorough manner. An important consideration when searching for statistically significant GO categories within a gene reporter list (our master-target test) is the choice of the reference (master) list of gene reporters from which the p-values for each GO category in the results are calculated. Some tools use the total set of genes in a genome as a reference (the master list). We do not think this is the best solution since the observed number of gene reporters for a specific GO category should be compared with the number of gene reporters that could appear if a random selection was taken from the list of all genes that was under study in the experiment.

In *e*GOn p-values can be shown for the whole GO tree and unlike most other tools several preset levels can be chosen and users can modify the tree as they like. In addition a result report view is accessible, showing only the GO nodes which satisfy a specific pre set p-value cutoff. Unique in the *e*GOn tool, we offer statistical tests for comparisons between gene reporter lists. The master-target test and mutually exclusive target-target test are both used in different variations in several programs today, but no other GO-tool, to our knowledge, offers tests for the intersecting target-target situation. However, the statistical test of FatiGO [37] is valid for the mutually exclusive target-target situation, and was in a simulation study found to preserve the test size when the gene reporter lists are of equal length [30]. Our intersecting target-target test is valid when the two gene reporter lists are intersecting, potentially constituting a useful test, since it offers the opportunity to compare gene reporter list for different experiments (as previously described by the heart diseases example). In this way both our target-target tests may answer questions not necessary answered by the standard master-target tests applied to most tools.

### Future plans

*GeneTools *was released in September 2005 and has steadily gained popularity since then. In October 2006 over 1 700 users from 60 countries were registered and over 4 000 gene reporter lists were submitted to the database. We plan to continue adding new features to *GeneTools*, including more information from external databases like e.g. Ensembl and OMIM. Furthermore, we hope to provide developers of other tools an extended version of our API and extend the export function to support SBML (systems biology markup language) [38] which will make more high-level analysis possible. We think the need for central and publicly available resources which curate biological data will only continue to grow and that *GeneTools *and similar tools will be essential for biologists and bioinformaticians to efficiently analyze genome-scale datasets. Today their main utility is for gene expression analysis, but in the future proteomic and SNP data need to be analyzed by similar tools. In addition, an important future use of annotation tools will be in systems biology approaches that are now evolving rapidly.

## Conclusion

*GeneTools *is a flexible and user friendly "all in one" annotation tool, where the users can rapidly extract gene annotation data for e.g. thousands of genes or clones at once. The user can add "user defined" GO annotation to gene products and all annotation information is stored in a database which can easily be shared with other users and exported in different formats. *e*GOn is the first tool that can perform hypothesis testing for three different situations, looking for over- or under-representation of GO categories between gene reporter lists.

## Availability and requirements

Project name: *GeneTools*

Project Homepage: 

Operating System: Platform independent

Programming Language: PHP

Underlying Database: mySQL

## Authors' contributions

VB initiated and coordinated the project and wrote the manuscript. AL co-initiated the project and together with AKS they supervised the project and were involved in drafting and reviewing the manuscript. ML, CCG and SL devised the statistical algorithms. FKJ, HB and HR designed and built the underlying database. LJ, HB and FKJ contributed equally in writing the program code and maintain the underlying database. FKJ, HB, and VB designed the *GeneTools *web interface. All authors read and contributed to revising the manuscript for intellectual content and approved the final manuscript.

## Supplementary Material

Additional file 1 eGOnv2_statistics.pdf. As supplementary materials a detailed description of the background for the statistical tests in *e*GOn is offered. Click here for file
