# Supplementary material for: GeneTools – application for functional annotation and statistical hypothesis testing
Source: BMC Bioinformatics. 2006 Oct 24;7:470. doi: 10.1186/1471-2105-7-470 (PMC1630634; doi:10.1186/1471-2105-7-470)
Supplement: Additional file 1 — eGOnv2_statistics.pdf. As supplementary materials a detailed description of the background for the statistical tests in eGOn is offered. [file 1471-2105-7-470-S1.pdf]

STATISTICAL HYPOTHESIS TESTING OF ASSOCIATION BETWEEN TWO GENE REPORTER LISTS WITHIN THE GO-HIERARCHY

Description of statistical tests in eGOn version 2.0

JULY 2006

1 BACKGROUND

Bioinformatics tools are needed to interpret functional information from large datasets obtained from genomic and proteomic high throughput technologies.

eGOn (explore Gene Ontology) is a tool that facilitates use of biological background knowledge in analysis of reporters (genes, probe sets, ESTs) selected from high throughput analysis like e.g microarray analysis. In addition to powerful graphical displays eGOn offers statistical hypothesis testing to assess the level of similarity between two different gene reporter lists, list A and list B.

2 THE NULL HYPOTHESIS

For a randomly chosen reporter and a given GO category G, define the following three events:

A = the reporter is on list A (e.g. has responded to treatment A)

B = the reporter is on list B (e.g. has responded to treatment B)

G = the reporter is a member of GO category G.

Denote by A^* the complementary event of A, B^* is the complementary event of B and G^* is the complementary event of G.

At the given GO category G, we are interested in investigating whether the probability of belonging to GO category G is different for reporters on gene reporter list A compared to reporters on gene reporter list B. I.e. for each reporter on list A, there is a probability $P(G|A)$ of belonging to GO category G, and for each reporter on list B, there is a probability $P(G|B)$ of belonging to GO category G. Under the null hypothesis these two probabilites are equal. We formulate the following null hypothesis and alternative hypothesis.

$$H_0 : P(G|A) = P(G|B) \text{ vs. } H_1 : P(G|A) \neq P(G|B) \quad (1)$$

$$H_0 : P(G|A) - P(G|B) = 0 \text{ vs. } H_1 : P(G|A) - P(G|B) \neq 0$$

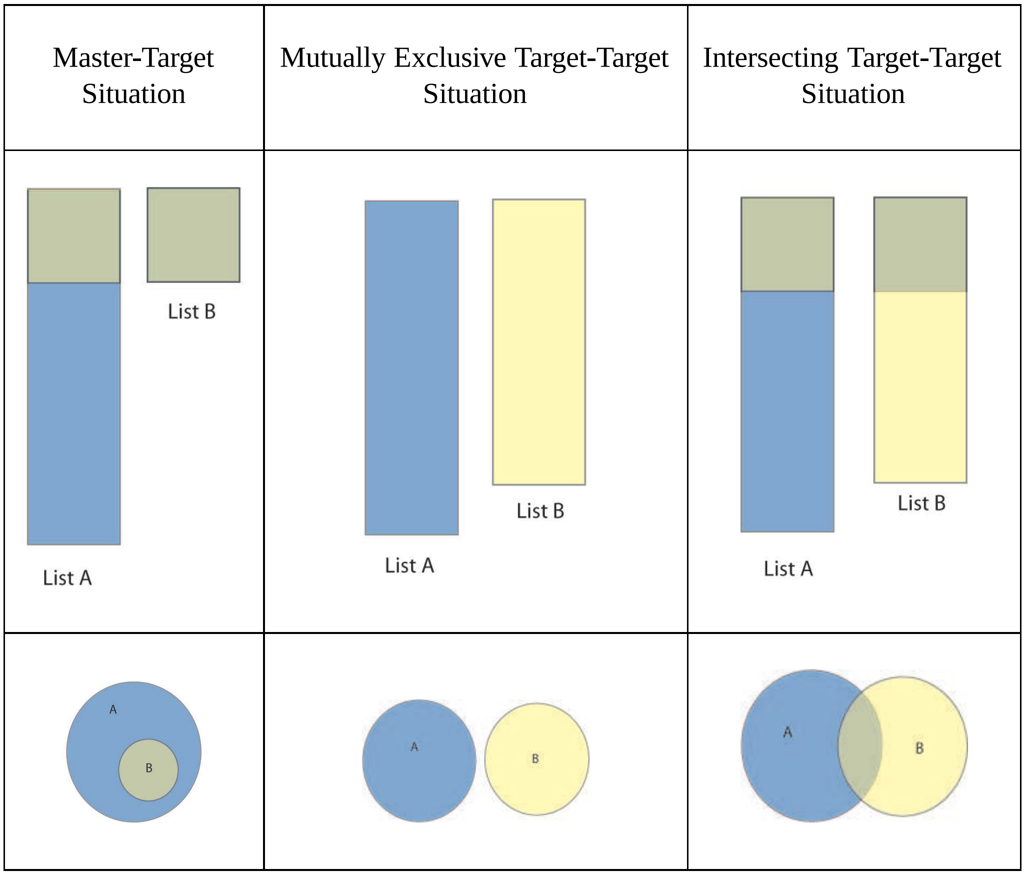

FIGURE 1: The three possible situations presented in the text.

Using the definition of conditional probability, the null hypotesis can be written equivalently as

$$\frac{P(A|G)}{P(B|G)} = \frac{P(A)}{P(B)}$$

This gives us the following additional interpretation. For a chosen GO category G, the ratio between the probability of membership on list A and membership on list B, is the same as the ratio between the probability of being a member of list A to the probability of being a member of list B in the whole GO-tree.

3 STATISTICAL TESTS

The tests outlined are based on the assumption that under the null hypothesis the genes on the lists (or subsets of the lists) act independently within each list.

Statistically we need to distinguish between three situations, to correctly handle the possible dependencies between gene lists A and B due to the fact that the same observational unit may be present on both lists. An illustration of these situations is given in Figure 1.

- Master-Target Situation: One of the two lists of genes compared is the list containing all genes present in the full experiment. (e.g. all genes assayed on the chip in a microarray experiment) see the left column of Figure 1.
- Mutually Exclusive Target-Target Situation: Two gene lists, A and B, are compared, and there are no genes that are on both lists, e.g. A is a list of genes associated with up-regulation and B is a list of genes associated with down-regulation. This situation is illustrated in the middle column of Figure 1.
- Intersecting Target-Target Situation: Two gene lists, A and B, are compared, and there exist genes that are on both lists, e.g. A is a list of genes associated with treatment A, and B is a list of genes associated with treatment B, see the right column of Figure 1.

3.1 Master-Target Situation

The gene reporter list A is now a master list, i.e. it contains all the reporters assayed. The gene reporter list B contains reporters of interest, e.g. reporters responding to treatment B. All the reporters on list B are also contained in list A.

Then testing the hypothesis (1) is equivalent to testing the following hypothesis

$$H_0 : P(G|A \cap B^*) = P(G|B) \text{ vs. } H_1 : P(G|A \cap B^*) \neq P(G|B) \quad (2)$$

A simple derivation of the equivalence of the null hypothesis in (1) and (2) is given in Appendix B of Günther, Langaas and Lydersen (2006).

For this situation we look at the 2×2 contingency table shown in Table 1, using the notation explained below. The term "number of genes" is read as "number of genes with annotation in the investigated GO-tree (molecular function, biological process or cellular component)".

- n_A: number of genes on the master list with annotation in the investigated GO-tree.
- n_B: number of genes on list B
- $n_{A\cap B^*}$: number of genes on the master list but not on list B. $n_A = n_B + n_{A\cap B^*}$
- n_G: number of genes from the master list in GO category G. $n_{G^*} = n_A - n_G$
- $X_{B\cap G}$: number of genes on the list B in GO category G. $X_{B\cap G^*}$ is the number of genes on the list B that is not in GO category G.
- $X_{(A\cap B^*)\cap G}$: number of genes on the master list but not on list B, that are in GO category G. Further $X_{(A\cap B^*)\cap G^*}$ is the number of genes on the master list but not on list B, that are not in GO category G.

Under the null hypothesis this is a hypergeometric situation. We have a total of n_A genes, where n_G genes are in the GO category we consider. The list B contains n_B genes and among these are $X_{B\cap G}$ in the GO category G. Then $X_{B\cap G}$ is hypergeometric distributed with parameters n_B, $n_{A\cap B^*}$ and n_G. That is,

	Event	G	G*	Total
1	B	$X_{B\cap G}$	$X_{B\cap G^*}$	n_B
2	$A\cap B^*$	$X_{(A\cap B^*)\cap G}$	$X_{(A\cap B^*)\cap G^*}$	$n_{A\cap B^*}$
	A	n_G	n_{G^*}	n_A

TABLE 1: *Situation when A is a master list and B is a general target list.*

$$P(X_{B\cap G} = x_{B\cap G} | n_B, n_{A\cap B^*}, n_G, n_G^*) = \frac{\binom{n_B}{x_{B\cap G}}\binom{n_{A\cap B^*}}{x_{A\cap B^*\cap G}}}{\binom{n_A}{n_G}}$$

By conditioning on the fixed marginals n_B, $n_{A\cap B^*}$, n_G and n_{G^*} we can use Fisher's exact test to calculate the p-value. The p-value, P, is then the sum of hypergeometric probabilities for all outcomes with probability equal or less to the observed hypergeometric probability, given the null hypothesis.

$$P = \sum_{p(y)\leq P(x_{B\cap G})} p(y)$$

Even when list B contains only a few reporters, this test is appropriate. A small p-value suggests that the probability of a reporter being in GO category G is different for the reporters on the list B compared to the reporters on the master list.

This test is called the Master-Target test in eGOn.

3.2 MUTUALLY EXCLUSIVE TARGET-TARGET SITUATION

When the gene reporter lists are mutually exclusive, $A\cap B = \emptyset$, there are no reporters that are on both list A and list B. This means that $A = A\cap B^*$ and $B = A^*\cap B$.

For this situation we look at the 2×2 contingency table shown in Table 2, using the notation explained below. Again, the term "number of genes" is read as "number of genes with annotation in the investigated GO-tree (molecular function, biological process or cellular component)".

- n_A: number of genes on list A (i.e. $A\cap B^*$).
- n_B: number of genes on list B (i.e. $A^*\cap B$).
- $n_{A\cup B}$: totalt number of genes on lists A and B.
- $n_{(A\cup B)\cap G}$: number of genes from the list A and B that are in GO category G. $n_{(A\cup B)\cap G^*} = n_{A\cup B} - n_{(A\cup B)\cap G}$.
- $X_{A\cap G}$: number of genes on the list A in GO category G. $X_{A\cap G^*}$ is the number of genes on the list A that are not in GO category G. $X_{A\cap G} + X_{A\cap G^*} = n_A$.
- $X_{B\cap G}$: number of genes on the list B in GO category G. $X_{B\cap G^*}$ is the number of genes on the list B that are not in GO category G. $X_{B\cap G} + X_{B\cap G^*} = n_B$.

	Event	G	G*	Total
1	A	$X_{A\cap G}$	$X_{A\cap G^*}$	n_A
2	B	$X_{B\cap G}$	$X_{B\cap G^*}$	n_B
	$A\cup B$	$n_{(A\cup B)\cap G}$	$n_{(A\cup B)\cap G^*}$	$n_{A\cup B}$

TABLE 2: *Situation with mutually exclusive lists A and B.*

Given the marginals n_A, n_B, $n_{(A\cup B)\cap G}$ and $n_{(A\cup B)\cap G^*}$ we look at the two independent binomial stochastic variables $X_{A\cap G}$ and $X_{B\cap G}$:

$$X_{A\cap G} \sim \text{binomial}(n_A, P(G|A))$$
$$X_{B\cap G} \sim \text{binomial}(n_B, P(G|B))$$

By conditioning on n_A, n_B and $(X_{A\cap G} + X_{B\cap G})$, $X_{A\cap G}$ is hypergeometric distributed with parameters n_A, n_B and $X_{A\cap G} + X_{B\cap G}$. That is,

$$P(X_{A\cap G} = x_{A\cap G}|X_{A\cap G} + X_{B\cap G}, n_A, n_B) = \frac{\binom{n_A}{x_{A\cap G}}\binom{n_B}{x_{B\cap G}}}{\binom{n_A+n_B}{x_{A\cap G}+x_{B\cap G}}}$$

We use Fisher's exact test to test the null hypothesis (1). We calculate the p-value, P, by adding the hypergeometric probabilities of all outcomes for all tables with the same marginals n_A, n_B and $X_{A\cap G} + X_{B\cap G}$ as the observed table, that have probability less or equal to the observed hypergeometric probability, given that the null hypothesis is true.

$$P = \sum_{p(y)\leq P(x_{A\cap G}|X_{A\cap G}+X_{B\cap G}, n_A, n_B)} p(y)$$

Fisher's exact test can be used even when there are only a few reporters on the lists. A small p-value suggests that the reporters on list A and B have different probabilites of being in GO category G.

This test is called the Target-Target test in eGOn.

3.3 INTERSECTING TARGET-TARGET SITUATION

When the gene reporter lists, A and B are overlapping, $A \cap B \neq \emptyset$, there exist genes that are on both list A and list B.

For this situation we look at the contingency table shown in Table 3, using the notation explained below. Again, the term "number of genes" is read as "number of genes with annotation in the investigated GO-tree (molecular function, biological process or cellular component)".

- $n_{A\cap B}$: number of genes both on list A and B.
- $n_{A\cap B^*}$: number of genes on list A and but not on list B.
- $n_{A^*\cap B}$: number of genes on list B, but not on list B.
- $n_{A\cup B}$: total number of genes on lists A and B.

- $n_{(A \cup B) \cap G}$: number of genes from the list A and B that are in GO category G. $n_{(A \cup B) \cap G} = n_{A \cup B} - n_{(A \cup B) \cap G}$.
- $X_{A \cap B \cap G}$: number of genes both on list A and B that also are in GO category G. $X_{A \cap B \cap G^*}$ is the number of genes both on list A and B that are not in GO category G.
- $X_{A \cap B^* \cap G}$: number of genes on list A, but not on list B that also are in GO category G. $X_{A \cap B \cap G^*}$ is the number of genes on list A, but not on B that are not in GO category G.
- $X_{A^* \cap B \cap G}$: number of genes on list B, but not on list A that also are in GO category G. $X_{A^* \cap B \cap G^*}$ is the number of genes on list B, but not on A that are not in GO category G.

In Günther et al. (2006) we have developed two conditional tests and translated one unconditional test from the work of Leisering, Alonzo and Pepe (2000). The test of Leisering et al. (2000) is implemented in eGOn and the following is a short outline of the test. For details the reader is referred to Günther et al. (2006). To simplify the presentation we use the notation in Table 4.

Based on a generalized linear model and generalized estimation equations Leisering et al. (2000) define a test statistic for large samples. For the case of two intersecting gene lists the test is based on the following test statistic:

$$T_{PPV} = \frac{((n_1 + n_2)(X_1 + X_3) - (n_1 + n_3)(X_1 + X_2))^2}{f(X_1, X_2, X_3, n_1, n_2, n_3)} \quad (3)$$

where

$$\begin{aligned} f(X_1, X_2, X_3, n_1, n_2, n_3) = \\ X_1(n_2 - n_3)^2 \left(1 - \frac{2X_1 + X_2 + X_3}{2n_1 + n_2 + n_3}\right)^2 \\ +X_2(n_1 + n_3)^2 \left(1 - \frac{2X_1 + X_2 + X_3}{2n_1 + n_2 + n_3}\right)^2 \\ +X_3(n_1 + n_2)^2 \left(1 - \frac{2X_1 + X_2 + X_3}{2n_1 + n_2 + n_3}\right)^2 \\ +(n_1 - X_1)(n_2 - n_3)^2 \left(\frac{2X_1 + X_2 + X_3}{2n_1 + n_2 + n_3}\right)^2 \\ +(n_2 - X_2)(n_1 + n_3)^2 \left(\frac{2X_1 + X_2 + X_3}{2n_1 + n_2 + n_3}\right)^2 \\ +(n_3 - X_3)(n_1 + n_2)^2 \left(\frac{2X_1 + X_2 + X_3}{2n_1 + n_2 + n_3}\right)^2 \end{aligned}$$

	Event	G	G^*	Total
1	$A \cap B$	$X_{A \cap B \cap G}$	$X_{A \cap B \cap G^*}$	$n_{A \cap B}$
2	$A \cap B^*$	$X_{A \cap B^* \cap G}$	$X_{A \cap B^* \cap G^*}$	$n_{A \cap B^*}$
3	$A^* \cap B$	$X_{A^* \cap B \cap G}$	$X_{A^* \cap B \cap G^*}$	$n_{A^* \cap B}$
	$A \cup B$	$n_{(A \cup B) \cap G}$	$n_{(A \cup B) \cap G^*}$	$n_{A \cup B}$

TABLE 3: Crosstabulation of events for intersecting gene lists

$A \cap B$	$A \cap B^*$	$A^* \cap B$
$o_1 = P(A \cap B)$	$o_2 = P(A \cap B^*)$	$o_3 = P(A^* \cap B)$
$p_1 = P(G \mid A \cap B)$	$p_2 = P(G \mid A \cap B^*)$	$p_3 = P(G \mid A^* \cap B)$
$X_1 = X_{A \cap B \cap G}$	$X_2 = X_{A \cap B^* \cap G}$	$X_3 = X_{A^* \cap B \cap G}$
$n_1 = n_{A \cap B}$	$n_2 = n_{A \cap B^*}$	$n_3 = n_{A^* \cap B}$

TABLE 4: Definition of o_i, p_i, x_i, and n_i for $i = 1, ..., 3$, used to simplify the presentation.

The p-value of the test can be calculated as:

$$P(\chi_1^2 \geq T_{PPV}) = 2(1 - \Phi(\sqrt{T_{PPV}})) \quad (4)$$

where Φ is the cumulative standard normal distribution.

4 MULTIPLE TESTING

Adjusted p-values are calculated using the step-up procedure of Benjamini and Hochberg (1995). Setting a cut-off at 0.05 for the adjusted p-value will control the False Discovery Rate (FDR) at level 0.05.

REFERENCES

BENJAMINI, Y. AND HOCHBERG, Y. (1995). Controlling the false discovery rate: A practical and powerful approach to multiple testing, *Journal of the Royal Statistical Society, Series B* 57(1): 289–300.

GÜNTHER, C.-C., LANGAAS, M. AND LYDERSEN, S. (2006). Statistical hypothesis testing of association between two lists of genes, Preprint Statistics 1/2006, Department of Mathematical Sciences, NTNU, available from http://www.math.ntnu.no/preprint/statistics/2006/.

LEISERING, W., ALONZO, T. AND PEPE, M. S. (2000). Comparisons of Predictive Values of Binary Medical Diagnostic Tests for Paired Designs, *Biometrics* 56: 345–351.
